# Supplementary material for: A Longitudinal Investigation of Blood Neurofilament Light Chain Levels in Chronic Cocaine Users
Source: Mol Neurobiol. 2023 Mar 31;60(7):3935–44. doi: 10.1007/s12035-023-03327-6 (PMC10224834; doi:10.1007/s12035-023-03327-6)
Supplement: Supplementary file 1 — ESM 1 [file 12035_2023_3327_MOESM1_ESM.docx]

| **Supplementary Table 1. Substance use patterns** | | | | | |
| --- | --- | --- | --- | --- | --- |
|  | ***Baseline*** | | | | |
| **Variables** | **Cocaine users**  **(N=35)** | **Healthy controls (N=35)** | **Test statistics** | **df** | ***P value*** |
| **Alcohol** |  |  |  |  |  |
| Times per week^a^ | 2.7 ± 2.4 | 2.5 ± 2.1 | U=597.0 | - | 0.855 |
| Grams per week^a,b^ | 132.2 ± 122.9 | 79.5 ± 73.9 | U=421.5 | - | **0.025** |
| Years of use | 18.6 ± 6.3 | 14.4 ± 6.8 | T=-2.6 | 68 | **0.010** |
| Estimated cumulative lifetime dose, Kg^b^ | 344.3 ± 509.1 | 68.1 ± 53.3 | U=285.0 | - | **<0.001** |
|  |  |  |  |  |  |
| **Cocaine** |  |  |  |  |  |
| Cocaine lifetime experience (yes/no) ^c^ | 35/0 | 5/30 | x2=52.5 | - | **<0.001** |
| Current cocaine dependence, DSM (yes/no) | 27/8 | - | - | - | - |
| Times per week^a^ | 2.4 ± 2.0 | - | - | - | - |
| Grams per week^a^ | 4.4 ± 7.6 | - | - | - | - |
| Years of use | 13.2 ± 6.7 | - | - | - | - |
| Estimated cumulative lifetime dose, g | 2,020 ± 2,276 | - | - | - | - |
| Abstinence period, days | 25.0 ± 49.6 | - | - | - | - |
| Cocaine_total_, pg/mg in hair^d^ | 33,102 ± 67,934 | - | - | - | - |
| Cocaine, pg/mg in hair | 24,752 ± 50,407 | - | - | - | - |
| Benzoylecgonine, pg/mg in hair | 7,769 ± 17,183 | - | - | - | - |
| Cocaethylene, pg/mg in hair | 647 ± 956 | - | - | - | - |
| Norcocaine, pg/mg in hair | 581 ± 988 | - | - | - | - |
| Levamisol pg/mg in hair | 2,962 ± 9,605 | - | - | - | - |
| Levamisole/cocaine ratio in hair | 0.17 ± 0.25 | - | - | - | - |
| Urine toxicology, (p/n)^e^ | 18/17 | - | - | - | - |
|  |  |  |  |  |  |
| **Nicotine** |  |  |  |  |  |
| Smoking (yes/no)^f^ | 29/6 | 27/8 | x2=0.09 | - | 0.759 |
| Cigarettes per week^a^ | 89.4 ± 70.2 | 60.1 ± 47.3 | U=421.0 | - | 0.113 |
| Years of use | 17.1 ± 7.6 | 11.8 ± 6.2 | U=321.0 | - | **0.004** |
|  |  |  |  |  |  |
| **Cannabis** |  |  |  |  |  |
| Cannabis lifetime experience, (yes/no) ^c^ | 33/2 | 31/4 | x2=0.73 | - | 0.393 |
| Times per week^a^ | 0.52 ± 1.19 | 0.21 ± 0.43 | U=445.5 | - | 0.357 |
| Grams per week^a^ | 0.47 ± 2.18 | 0.08 ± 0.17 | U=437.0 | - | 0.298 |
| Years of use | 13.3 ± 9.7 | 7.6 ± 5.8 | U=334.0 | - | **0.017** |
| Estimated cumulative lifetime dose, g | 3,053 ± 5,287 | 138 ± 382 | U=221.5 | - | **<0.001** |
| Abstinence period, days | 1,225 ± 2,554 | 1,326 ± 2,344 | U=473.0 | - | 0.605 |
| THC, pg/mg in hair | 32.0 ± 105.4 | 12.9 ± 54.8 | U=498.5 | - | 0.159 |
| CBD, pg/mg in hair | 6.3 ± 15.5 | 1.4 ± 4.5 | U=441.5 | - | **0.034** |
| Urine toxicology, (p/n)^e^ | 5/30 | 2/33 | x2=1.4 | - | 0.232 |
|  |  |  |  |  |  |
| **MDMA** |  |  |  |  |  |
| MDMA lifetime experience, (yes/no)^c^ | 31/4 | 9/26 | x2=28.2 | - | **<0.001** |
| Grams per week^a^ | 0.01 ± 0.04 | 0.001 ± 0.002 | U=133.0 | - | 0.815 |
| Years of use | 8.4 ± 8.2 | 1.5 ± 1.5 | U=57.0 | - | **0.007** |
| Estimated cumulative lifetime dose, g | 50.4 ± 169.2 | 0.1 ± 0.2 | U=39.0 | - | **0.001** |
| Abstinence period, days | 1,405 ± 2,319 | 1,269 ± 2,754 | U=134.0 | - | 0.859 |
| MDMA, pg/mg in hair | 2,289 ± 9,332 | 3.7 ± 13.6 | U=344.5 | - | **<0.001** |
|  |  |  |  |  |  |
| **Amphetamine** |  |  |  |  |  |
| Amphetamine lifetime experience, (yes/no)^c^ | 25/10 | 4/31 | x2=26.0 | - | **<0.001** |
| Grams per week^a^ | 0.02 ± 0.03 | - | - | - | - |
| Years of use | 8.1 ± 7.0 | 0.3 ± 0.3 | U=17.5 | - | **0.040** |
| Estimated cumulative lifetime dose, g | 132.1 ± 293.1 | 0.1 ± 0.2 | U=19.0 | - | 0.050 |
| Abstinence period, days | 853 ± 903 | 1407 ± 1920 | U=46.5 | - | 0.825 |
| Amphetamine, pg/mg in hair | 32 ± 367 | 0.7 ± 4.2 | U=8.0 | - | 0.262 |
| Urine toxicology, (p/n)^e^ | 0/35 | 0/35 | - | - | - |
| Table reports counts or means ± standard deviations. Significant group differences are shown in bold. t = Student t-test; x2 = Pearson chi-square; U = Mann-Whitney test.  Here we specifically reported the most prevalent substances and metabolites.  ^a^ Average use of the current consumption period; ^b^ Pure alcohol estimation; ^c^ Self-report: Have you ever consumed this substance, at least once, in your life?; ^d^ Cocaine_total_ (= Cocaine + Benzoylecgonine + Norcocaine) is a more robust procedure for discrimination between incorporation and contamination of hairs (Hoelzle et al., 2008). ^e^ Urine toxicology (negative/positive) are based on cut-off value for cocaine = 150 pg/ml and for tetrahydrocannabinol = 50 pg/ml (Substance Abuse and Mental Health Services Administration, 2008); ^f^ Subjects are considered smokers when they smoke >= 7 cigarettes/week.  Abbreviations: CBD, cannabinoid; MDMA, 3,4-Methyl​enedioxy​methamphetamine; THC, Tetrahydrocannabinol. | | | | | |

**Supplementary Table 2. Correlations of NfL levels with sociodemographic and clinical characteristics at baseline**

| **Variables** | **Cocaine users** | | | **Healthy controls** | | | **Overall** | | |
| --- | --- | --- | --- | --- | --- | --- | --- | --- | --- |
|  | ***ρ*** | ***P value*** | **N** | ***ρ*** | ***P value*** | **N** | ***ρ*** | **P value** | **N** |
| Age, years | 0.32 | 0.065 | 35 | 0.51 | **0.002** | 35 | 0.48 | **<0.001** | 70 |
| Sex (f/m) | 0.24 | 0.175 | 35 | 0.31 | 0.075 | 35 | 0.23 | 0.054 | 70 |
| BMI, kg/m2 | -0.33 | 0.053 | 35 | -0.43 | **0.010** | 35 | -0.30 | **0.013** | 70 |
| Education, years | 0.13 | 0.462 | 35 | -0.14 | 0.420 | 35 | -0.09 | 0.471 | 70 |
| Family psychiatric history (yes/no) | -0.11 | 0.544 | 35 | - | - | - | - | - | - |
| ADHD_SR, score | -0.05 | 0.763 | 35 | 0.25 | 0.156 | 35 | 0.15 | 0.150 | 70 |
| ADHD DSM (yes/no) ^a^ | -0.06 | 0.753 | 35 | 0.08 | 0.661 | 35 | 0.01 | 0.931 | 70 |
| History of MDD DSM (yes/no) ^b^ | -0.18 | 0.311 | 35 | -0.13 | 0.452 | 35 | -0.03 | 0.828 | 70 |
| CTQ, score | -0.07 | 0.686 | 35 | -0.15 | 0.393 | 35 | -0.04 | 0.736 | 70 |
| BDI, score | -0.10 | 0.573 | 35 | 0.15 | 0.377 | 35 | 0.10 | 0.406 | 70 |
| Table reports correlations between NfL levels and sociodemographic and clinical characteristics in each group. Significant group differences are shown in bold. *ρ =* Spearman's rank correlation coefficient.  ^a^ Cut-off according to DSM-IV criteria as assessed by the ADHD-SR questionnaire; ^b^ Cut-off according to DSM-IV criteria as assessed by SCID-I interview;  Abbreviations: NFL: ADHD, Attention deficit hyperactivity disorder; BDI: Beck Depression Inventory; BMI: Body Mass Index; CTQ: Childhood Trauma Questionnaire; MDD: major depressive disorder; Neurofilament Light Chain. | | | | | | | | | |

**Supplementary Table 3. Rank correlations of NfL levels with substance use pattern in cocaine users.**

| **Variables** | **Cocaine Users (N=35)** | | |
| --- | --- | --- | --- |
|  | ***ρ*** | ***P value*** | **N** |
| **Alcohol** |  |  |  |
| Grams per week^a,b^ | 0.03 | 0.879 | 35 |
|  |  |  |  |
| **Cocaine** |  |  |  |
| Cocaine_total_, pg/mg in hair ^c^ | 0.36 | **0.032** | 35 |
| Cocaine, pg/mg in hair | 0.35 | **0.041** | 35 |
| Benzoylecgonine, pg/mg in hair | 0.35 | **0.038** | 35 |
| Cocaethylene, pg/mg in hair | 0.36 | **0.033** | 35 |
| Norcocaine, pg/mg in hair | 0.29 | 0.094 | 35 |
| Levamisol pg/mg in hair | 0.18 | 0.293 | 35 |
| Levamisole/cocaine ratio in hair | 0.20 | 0.912 | 35 |
|  |  |  |  |
| **Cannabis** |  |  |  |
| THC, pg/mg in hair | 0.27 | 0.121 | 35 |
| CBD, pg/mg in hair | 0.01 | 0.949 | 35 |
|  |  |  |  |
| **MDMA** |  |  |  |
| MDMA, pg/mg in hair | 0.10 | 0.562 | 35 |
|  |  |  |  |
| **Amphetamine** |  |  |  |
| Amphetamine, pg/mg in hair | -0.03 | 0.848 | 35 |
| Table reports correlations between NfL levels and substance use variables. Significant group differences are shown in bold. *ρ =* Spearman's rank correlation coefficient.  ^a^ Average use of the current consumption period; ^b^ Pure alcohol estimation; ^c^Cocaine_total_ (= Cocaine + Benzoylecgonine + Norcocaine) is a more robust procedure for discrimination between incorporation and contamination of hairs (Hoelzle et al., 2008); | | | |

**Supplementary Table 4. Correlations of change of NfL levels (Δ_NfL_) with change of substance use pattern.**

| **Variables (Δ)** | **Cocaine Users (N=26)** | | |
| --- | --- | --- | --- |
|  | ***ρ*** | ***P value*** | **N** |
| **Alcohol** |  |  |  |
| Grams per week^a,b^ | -0.02 | 0.935 | 26 |
|  |  |  |  |
| **Cocaine** |  |  |  |
| Cocaine_total_, pg/mg in hair ^c^ | **0.58** | **0.002** | **26** |
| Levamisol pg/mg in hair | 0.05 | 0.819 | 26 |
|  |  |  |  |
| **Cannabis** |  |  |  |
| THC, pg/mg in hair | 0.37 | 0.064 | 26 |
| CBD, pg/mg in hair | 0.37 | 0.063 | 26 |
|  |  |  |  |
| **MDMA** |  |  |  |
| MDMA, pg/mg in hair | 0.11 | 0.589 | 26 |
|  |  |  |  |
| **Amphetamine** |  |  |  |
| Amphetamine, pg/mg in hair | 0.05 | 0.802 | 26 |
| Table reports correlations between change of NfL levels and change of substance use variables over the follow-up period (Δ_X_=[value substance X at T2]- [value substance X at T1]). Significant group differences are shown in bold. *ρ =* Spearman's rank correlation coefficient.  ^a^ Average use of the current consumption period; ^b^ Pure alcohol estimation; ^c^Cocaine_total_ (= Cocaine + Benzoylecgonine + Norcocaine) is a more robust procedure for discrimination between incorporation and contamination of hairs (Hoelzle et al., 2008); | | | |
